# Involvement of Interleukin-17A-Induced Hypercontractility of Intestinal Smooth Muscle Cells in Persistent Gut Motor Dysfunction

**DOI:** 10.1371/journal.pone.0092960

**Published:** 2014-05-05

**Authors:** Hirotada Akiho, Yohei Tokita, Kazuhiko Nakamura, Kazuko Satoh, Mitsue Nishiyama, Naoko Tsuchiya, Kazuaki Tsuchiya, Katsuya Ohbuchi, Yoichiro Iwakura, Eikichi Ihara, Ryoichi Takayanagi, Masahiro Yamamoto

**Affiliations:** 1 Department of Medicine and Bioregulatory Science, Graduate School of Medical Sciences, Kyushu University, Fukuoka, Japan; 2 Department of Gastroenterology, Kitakyushu Municipal Medical Center, Fukuoka, Japan; 3 Tsumura Research Laboratories, Tsumura & Co., Ibaraki, Japan; 4 Division of Laboratory Animal, Research Institute for Biomedical Science, Tokyo University of Science, Chiba, Japan; 5 Core Research for Evolutional Science and Technology (CREST), JST, Saitama, Japan; University of Nevada School of Medicine, United States of America

## Abstract

**Background and Aim:**

The etiology of post-inflammatory gastrointestinal (GI) motility dysfunction, after resolution of acute symptoms of inflammatory bowel diseases (IBD) and intestinal infection, is largely unknown, however, a possible involvement of T cells is suggested.

**Methods:**

Using the mouse model of T cell activation-induced enteritis, we investigated whether enhancement of smooth muscle cell (SMC) contraction by interleukin (IL)-17A is involved in postinflammatory GI hypermotility.

**Results:**

Activation of CD3 induces temporal enteritis with GI hypomotility in the midst of, and hypermotility after resolution of, intestinal inflammation. Prolonged upregulation of IL-17A was prominent and IL-17A injection directly enhanced GI transit and contractility of intestinal strips. Postinflammatory hypermotility was not observed in IL-17A-deficient mice. Incubation of a muscle strip and SMCs with IL-17A *in vitro* resulted in enhanced contractility with increased phosphorylation of Ser19 in myosin light chain 2 (p-MLC), a surrogate marker as well as a critical mechanistic factor of SMC contractility. Using primary cultured murine and human intestinal SMCs, IκBζ- and p38 mitogen-activated protein kinase (p38MAPK)-mediated downregulation of the regulator of G protein signaling 4 (RGS4), which suppresses muscarinic signaling of contraction by promoting inactivation/desensitization of Gα_q/11_ protein, has been suggested to be involved in IL-17A-induced hypercontractility. The opposite effect of L-1β was mediated by IκBζ and c-jun N-terminal kinase (JNK) activation.

**Conclusions:**

We propose and discuss the possible involvement of IL-17A and its downstream signaling cascade in SMCs in diarrheal hypermotility in various GI disorders.

## Introduction

GI motility disorders, such as GI infection, IBD, ileus, achalasia and functional gastrointestinal disease, have been associated with immune activation [1]–[5]. Patients after acute bacterial gastroenteritis and those with IBD in remission, typically Crohn's disease (CD), often develop symptoms of irritable bowel syndrome (IBS), termed post-infectious IBS (PI-IBS) and IBD-IBS, respectively [6], [7]. The key features of these disorders include pain and usually diarrheal symptoms with minimal or no evident intestinal inflammation [6]. Previous studies suggest that infiltrating T lymphocytes in the intestinal muscle layer play an important role in motility dysfunction. The critical role of T helper type 2 (Th2) signaling driven by transcription factor Stat6 and T cell cytokines, such as IL-4 and IL-13, have been shown in post-infectious gut hypermotility by using experimental nematode infection models [8]–[10]. However, in the pathogenesis of CD, IL-12/Th1 and IL-23/Th17 pathways, rather than the Th2 pathway, are believed to be predominantly involved [1], [11]. Proinflammatory and Th1 cytokines such as tumor necrosis factor α (TNF-α), IL-1β and interferon-γ (IFN-γ) are known to induce strong hypomotility by directly decreasing the contractility of intestinal SMCs [12]–[14]. Thus, the mechanism underlying hypermotility in IBD-IBS is still unknown. Here, using a mouse model of T cell activation-induced enteritis, we show IL-17A may be involved in GI hypermotility in the model by inducing hypercontractility in SMCs. Although the model may not directly reflect the pathological situations of clinical IBD nor IBS, and although IL-17A is not essential for induction of inflammation in this model, the present findings address the possibility that IL-17A may modulate GI motility in the healing stage after intestinal inflammation. Furthermore, we analyzed the detailed molecular mechanisms, which indicate the contractile response of SMCs is regulated *via* crosstalk between mitogen-activated protein kinases and IκBζ-mediated modulation of the regulator of G protein signaling 4 (RGS4).

## Materials and Methods

### Mice

BALB/c mice were purchased from Japan SLC, Inc. (Hamamatsu, Japan). IL-17A-deficient mice (Il17a^tm1Yiw^) of BALB/c strain were described previously [15]. This study was carried out in strict accordance with the recommendations in the Guide for the Care and Use of Laboratory Animals of the National Institutes of Health. The protocol was approved by the Committee on the Ethics of Animal Experiments of Tsumura Research Laboratories (Permission Number: 08–210).

### Chemicals

Culture media and supplements were obtained from Lifescience Technologies (Carlsbad, CA.). Various MAPK and NFκB inhibitors were purchased from Calbiochem (San Diego, CA). Other reagents were from Sigma-Aldrich (St. Louis, MO) unless otherwise stated.

### Cytokines and antibodies

Recombinant human and murine IL-17A, IL-1β and IL-4 and an IL-17R-Fc-Chimera antibody were obtained from R&D systems (Minneapolis, MN). Antibodies to CD3 (αCD3, clone 145-2C11, mouse monoclonal, BD Bioscience, San Jose, CA), IL-17R (mouse monoclonal, R&D systems), NFκB p65 (rabbit monoclonal, Cell Signaling Technology, Danvers, MA), total myosin light chain-2 (t-MLC, rabbit polyclonal, Cell Signaling Technology), phospho-myosin light chain 2 (Serine 19) (p-MLC, mouse monoclonal, Cell Signaling Technology), RGS4 (rabbit polyclonal, LifeSpan Biosciences, Seattle, WA), α-smooth muscle actin (rabbit monoclonal, Novus Biologicals, Littleton, CO), and MAPK antibodies (rabbit polyclonal, Cell Signaling Technology) were used.

### αCD3-induced motility disorder model

Male 8- to 10-week old mice were injected with 12.5 µg of αCD3 intraperitoneally. 1, 3 and 7 days after αCD3 or PBS injection, we evaluated the histology, GI transit and contractility of SI muscle strip and isolated SMCs. The cytokine protein and mRNA levels in whole tissue and isolated longitudinal muscle (LM) layer were also determined. For estimation of GI transit, mice were orally administered with 200 µL of fluorescein-labelled dextran of 70,000 MW (FD70; Invitrogen, Carlsbad, CA) and the GI tract was excised after 30 min. Fluorescence was visualized and quantified using the G-box system (Syngene, Cambridge, UK) [16] and the geometric center was calculated using the formula: Σ(% FD70 per segment ×segment number)/100. In some experiments, murine IL-17A (10 µg/mouse/day) was intraperitoneally injected for three days and GC was measured on day 5.

### Contractility assays

Muscle strip (MS): Excised small intestinal segments or peeled LM strips were placed in an organ bath filled with oxygenated (95% O_2_ and 5% CO_2_) Krebs-Henseleit solution. Tension generated along the longitudinal axis of the strip was recorded with an isometric tension transducer + digital bridge amplifier (World Precision Instruments, Sarasota, FL), and visualized with the Data Acquisition System (Acknowledge-MP100 system; BIOPAC Systems, Goleta, CA). Contractile force (g) of each MS was normalized by their length in place of the conventional method using tissue weight or section area because 1) histochemistry revealed that the morphology of SMC and MS at 1 week after αCD3 injection returned to normal (Figure S1), 2) accordingly, the distribution of the plots of length and weight of intestinal segments and their correlation coefficient were similar between αCD3-treated and PBS treated groups (data not shown), 3) the correlation between length and wet weight was significant and strong in the present experimental setting (data not shown). The dose-related effects of carbamylcholine chloride, (CCh; Sigma-Aldrich), (1 nmol/L–1 mmol/L) was examined cumulatively [17]. In some experiments, LM strips were incubated with 1 ng/ml/day of mouse recombinant IL-17A for 3 days in medium 199 (M199, Life Technologies) containing 1% antibiotics-antimycotics (Sigma-Aldrich).

Isolated murine SMC: Excised LM from mouse small intestine was dispersed in HEPES buffer containing 1 mg/ml of collagenase, BSA and trypsin inhibitor (Sigma-Aldrich) at 31°C [8]. The cells were washed and then harvested through a 210-µm nylon mesh. In some cases, SMCs were incubated with recombinant mouse IL-17A for 24 hr in DMEM (Life Technologies) containing 1% antibiotics-antimycotics. SMCs were stimulated by the addition of CCh for 30 seconds and fixed with 1% acrolein. The cells were further stained with DAPI and phalloidin and the length of SMCs, which maintain normal morphological integrity of actin and nucleus was measured using a cell-imaging system Celaview RS100 (Olympus, Tokyo, Japan). The contractile response was taken as the ratio of the average length of SMCs exposed to CCh to that of SMCs exposed to PBS.

Cultured human colonic and murine SI SMCs: The cells were cultured in the temperature-responsive 96 well culture plates (UpCell plates, CellSeed Inc., Tokyo, Japan) at 37°C. The confluent cell layers were incubated with various concentrations of IL-17A for 4–5 days. After addition of CCh (10^−11^ M), the plates were incubated at room temperature, which induced the detachment of the cell layer from the bottom of the well by altering the hydrophobicity of polymers covalently bound to the plate surface. The cell layer was fixed with 1% acrolein and the contractility was estimated by measuring the area of shrinking cell layers using BIOREVO BZ-9000 (Keyence, Osaka, Japan).

### Quantitation of mRNA and protein of cytokines

Total RNA was prepared using an RNeasy Lipid Tissue Mini Kit (QIAGEN GmbH, Hilden, Germany). One µg of totalRNA was reverse transcribed by High Capacity cDNA Reverse Transcription Kits (Life Technologies) and the cDNA samples were analyzed by real-time PCR by using TaqMan Gene Expression Master Mix (Life Technologies). Real-time PCR was monitored using an ABI Prism 7900 HT (Life Technologies) with a TaqMan gene expression assay probe. Ubiquitin C was analyzed as an internal control. For quantitation of cytokines, supernatants from tissue homogenates were analyzed using a Bio-Plex Suspension Array System with Millipex Mouse Cytokine Panel 1 and Bio-Plex Pro Mouse Cytokines Panel Group 1 (Bio-Rad Laboratories, Hercules, CA). Results were corrected for protein concentration, which was measured using Bradford protein assay kit (Bio-Rad Laboratories).

### Microarray

Total RNA was prepared using an RNeasy mini kit (QIAGEN). The quality of the total RNA was assessed using a Bioanalyzer (Agilent Technologies, Palo Alto, CA). RNAs were labeled with the Agilent Quick Amp labeling kit (Agilent Technologies) and hybridized Agilent whole genome oligo microarray (8×60 k, Agilent Technologies). The microarray data sets were normalized by Agilent GeneSpring GX 10.0 using the Agilent FE one-color scenario (global normalization). Data were deposited to Gene Expression Omnibus (http://www.ncbi.nlm.nih.gov/geo/) and are available under the series ID: GSE39573.

### Cell culture

Murine LM was digested in the buffer containing 0.1% type II collagenase and 0.1% soybean trypsin inhibitor (Sigma-Aldrich) and the dispersed cells were plated in type IV-collagen coated plates (BD Bioscience) in HuMedia-SG2 (Kurabo, Osaka, Japan). After 9 days of culture, medium was replaced with M199 containing antibiotics-antimycotics. After an additional 24 hr of culture, cytokines and/or inhibitors were added. Human colonic SMCs were purchased from ScienCell Research Laboratories (Carlsbad, CA) and cultured according to the supplier's protocol. Changes of medium and treatment with cytokines/inhibitors were performed as described above. SN50 (Enzo Biochemicals, Farmingdale, NY), and various siRNAs (Dharmacon siRNA; ThermoFisher Scientific, Lafayette, CO) were examined.

### Immunoblot analysis

LM strips and cultured SMCs were homogenized in the lysis buffer containing protease and phosphatase inhibitor cocktails (ThermoFisher Scientific) and the amount of protein was measured using a RC-DC-protein assay kit (BioRad). Samples were separated by SDS-PAGE, transferred to Immobilon-P membranes (Millipore, Bedford, MA), and probed with primary antibodies and finally, with fluorescein-conjugated secondary antibodies (Life Technologies). The bands were visualized using an enhanced chemiluminescence (ECL) system (Pierce, Rockford, IL). Phosphorylation of serine 19 in myosin light chain 2 (p-MLC) was evaluated by calculating the ratio of the band intensity of p-MLC to that of t-MLC, which was quantified on a separate immunoblot in which the same amount of sample was loaded (Figure 5G). For the experiments shown in Figures 3B and 7B, an additional normalization of saline- and cytokine-treated samples to non-treated naïve samples (Control) was performed. This additional normalization was required because the basal levels of p-MLC/t-MLC fluctuated among the individual mice and/or separate experiments.

### Immunostaining and imaging analysis

Immunohistochemistry and immunocytochemistry were performed on frozen-sections of MS post-fixed by acetone, and cultured SMC on type IV collagen-coated slides/dishes, respectively, using antibodies to NFκB p65 and RGS4. Samples were viewed using a laser-scanning confocal microscope FV1000 (Olympus, Tokyo, Japan) and a cell-imaging system Celaview RS100 (Olympus). Three dimensional reconstitution of confocal images was performed using the FV1000's built-in program. Nuclear translocations of NFκB p65 protein was assessed by measuring the intensity of p65 immunosignal in the nuclei which were picked up automatically by Celaview.

### RGS4 activity

RGS4 translocation to the plasma membrane from the cytosol was evaluated by two methods, immunoblotting and imaging analysis. Subcellular fractions were prepared using ProteoExtract Subcellular Proteome Extraction Kit (Calbiochem) from peeled mouse LM strips and cultured human/mouse SMCs. Cytosolic and nuclear fractions and plasma membrane fractions were electrophoresed and analyzed by immunoblotting. The ratio of plasma membrane RGS4 to the cytosolic/nuclear RGS4 was then calculated. Alternatively, because the amount of RGS4 protein was too low for immunoblotting during the early stage of SMC culture, RGS4 translocation was quantified by imaging analysis using Celaview. Percentage of the cells having the “punctate RGS signal” to the total number of cells were counted manually in a blind fashion.

### MAPK activities

Screening of MAPK activities were performed as follows: SMCs cultured with cytokines for 4 days were lysed and the activities of 24 MAPKs then measured using a ProteomeProfiler kit (R&D Systems). Activation of p38 MAPK and JNK was measured by immunoblot as the ratio of the phosphorylated band to the total band.

### Statistical analysis

Data are expressed as mean ± s.e.m. We performed an unpaired Student's t-test to analyze differences between two groups of mice. The difference of dose-responsive effect was analyzed by repeated measure ANOVA. Among three or more groups of mice, an unpaired Student's t-test corrected using the Bonferroni method or the closed testing procedure, were performed to determine statistical significance. P<0.05 was considered a significant difference.

## Results

### Hypermotility after resolution of T-cell-activation-induced eneteropathy was mediated by IL-17A

**Figure 1:**
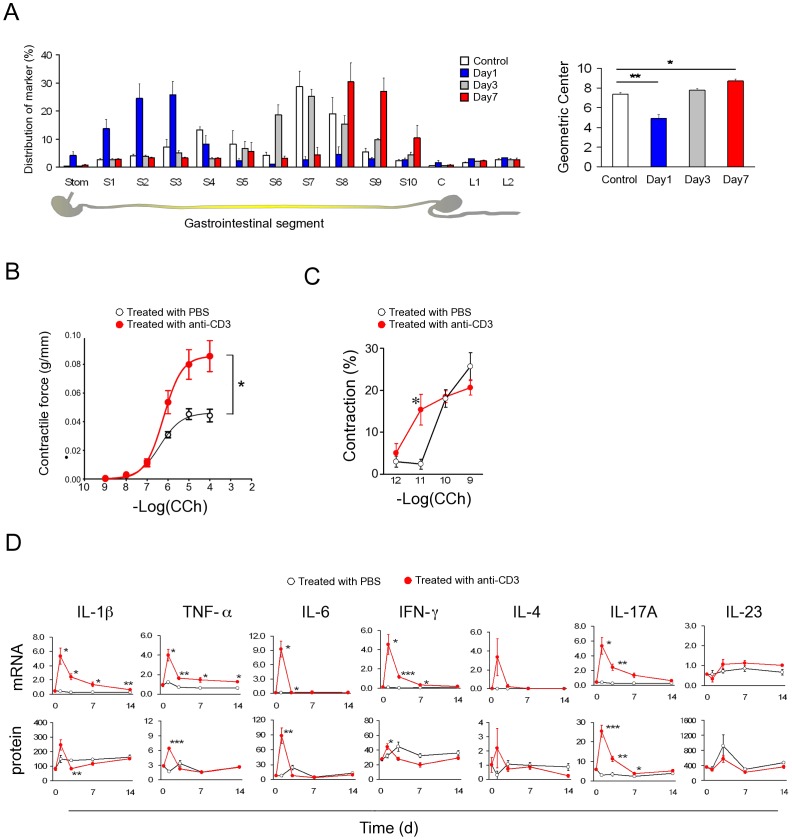
T cell activation induces GI hypermotility after resolution of inflammation, with sustained upregulation of IL-17A protein. (**A**) GI transit distribution histograms and calculated geometric center (GC) values from the histograms. GC has been frequently and reliably used to estimate GI transit. Stom, stomach; S1–S10, small intestine segments 1–10; C, caecum; L1–2, Large intestine segments 1–2. Control (PBS-treated), day 1, 3 and 7 after αCD3 injection (n = 3 for each group of mice). (**B**) Carbachol (CCh)-stimulated dose-response curves of contractile response of SI strips prepared from day 7 with or without αCD3 treatment (n = 7). CCh was added cumulatively. (**C**) CCh-stimulated dose-response curves of contractile response of SI longitudinal SMCs prepared from day 7 with or without αCD3 treatment. The respective concentration of CCh was added separately (n = 4). (**D**) Time-dependent changes of mRNA and protein levels of various Th-cytokines in the intestinal tissue measured by quantitative RT-PCR and Bio-plex assay, respectively (n = 4∼8). Numerical data represent means ± s.e.m. *P<0.05, **P<0.01 versus control/PBS-treated, repeated measure ANOVA (**B**), Student's t-test (**A, C, D**) with Bonferroni correction for multiple comparison (**A**).

**Figure 2:**
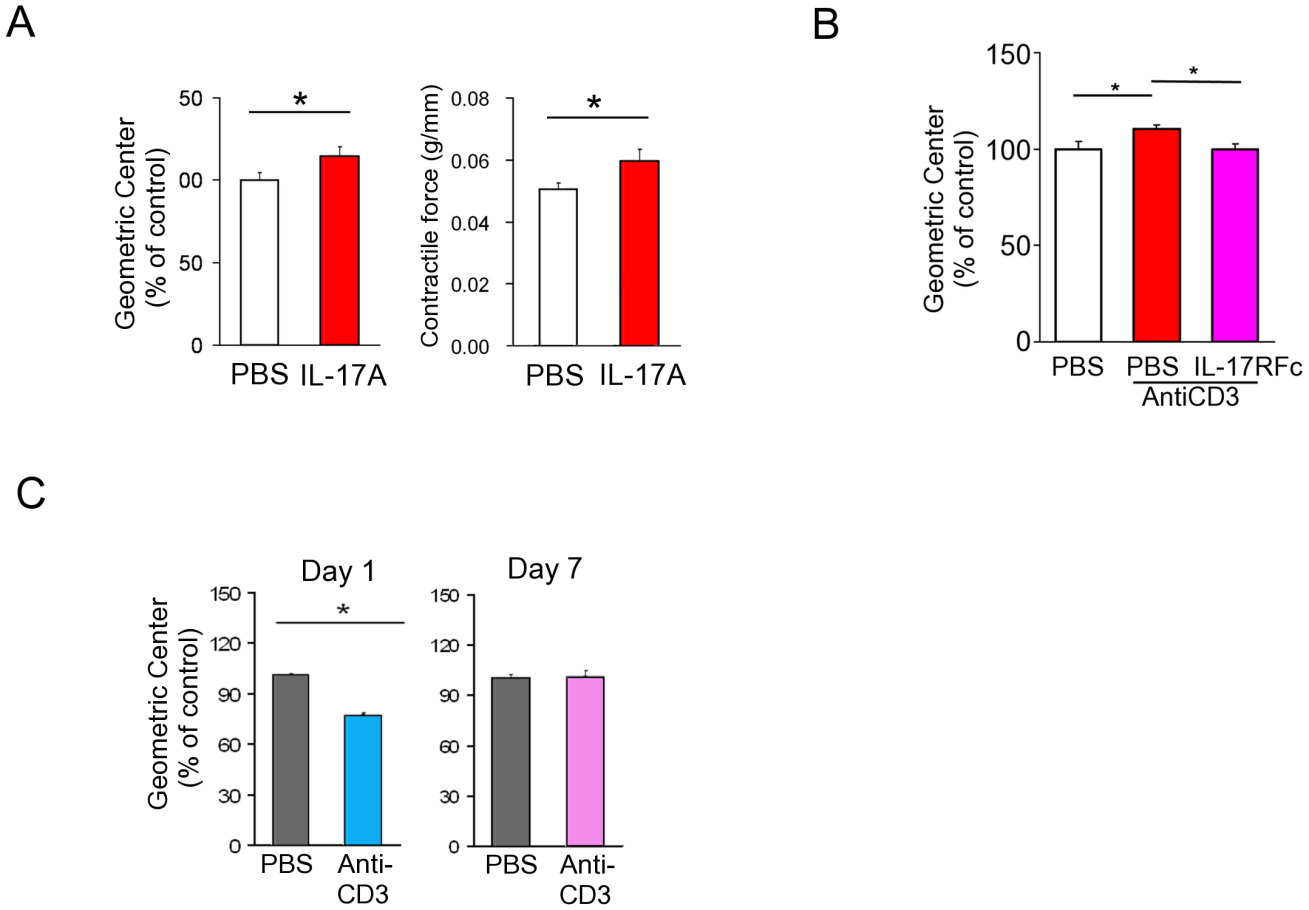
A possible involvement of IL-17A in αCD3-induced hypermotility. (**A**) Calculated GCs (left panel) and contractile response induced by 10^−5^ M CCh (right panel) of IL-17A-injected mice (n = 11∼15). Saline or IL-17A (10 µg/mouse/day) was intraperitoneally injected three times. GC was measured on day 5. SI strips was isolated on day 5 and the contractility was immediately measured. (**B**) Calculated GCs of αCD3-injected mice in the recovery phase. The effect of IL-17R-FcChimera (twice i.p. at 1.5 and 3 hrs after αCD3-injection; n = 13–15) was demonstrated. (**C**) Calculated GCs of control and αCD3-injected IL-17 KO mice in the day 1 inflammatory phase (n = 3) and in the day 7 recovery phase (n = 11∼13). Numerical data represent means ± s.e.m. *P<0.05 versus control/PBS-treated, Student's t-test (A-C) with Bonferroni correction for multiple comparison (**B**).

Mice injected with a T-cell-activating anti-CD3 antibody (αCD3) develop a transient diarrheal illness [18]–[20]. The small intestinal (SI) tissue damage in the early phase (1–3 days after αCD3 injection) is characterized by enterocyte apoptosis, epithelial damage and villous atrophy which had recovered by day 7 in terms of histology (Figures S1A, B). However, αCD3-treated mice on day 1 (the inflammatory phase) showed hypomotility, but then displayed hypermotility on day 7, in the recovery phase (Figure 1A). SI strips isolated on day 7 also showed enhanced contractile responses upon stimulation by an acetylcholine (ACh) analog, carbamylcholine chloride (CCh) (Figures 1B). The contractile response was not abrogated by tetrodotoxin treatment (data not shown). Accordingly, SMCs isolated on day 7 also showed enhanced CCh-induced contractile responses (Figures 1C). The antibody induced only a modest elevation in the level of SI TNF-α, IL-1β, IFN-γ, IL-4 and IL-23 proteins while IL-6 and IL-17A increased by several fold. Furthermore, a significantly higher level of IL-17A was observed on day 7 (Figure 1D). Real-time RT-PCR analysis also indicated that IL-17A is predominantly induced by αCD3 treatment among IL-17A∼F subtypes (data not shown). We therefore investigated whether IL-17A is involved in the aberration of gastrointestinal transit in this model. Intraperitoneal injection of IL-17A significantly enhanced GI transit and contractility of MS (Figure 2A). Treatment with IL-17R Fc-chimera, an efficient antagonistic chimeric antibody to IL-17A–IL-17 receptor interaction, abrogated the αCD3-induced acceleration of GI transit (Figure 2B). In addition, In IL-17A KO mice, while the hypomotility of GI transit in the inflammatory phase was shown, hypermotility in the recovery phase disappeared (Figure 2C). There were no apparent difference in the enteropathy of SI between IL-17A KO and wild-type mice (Figures S1A–C). These data suggest that IL-17A is responsible for post-inflammatory hypermotility in the GI tract.

**Figure 3:**
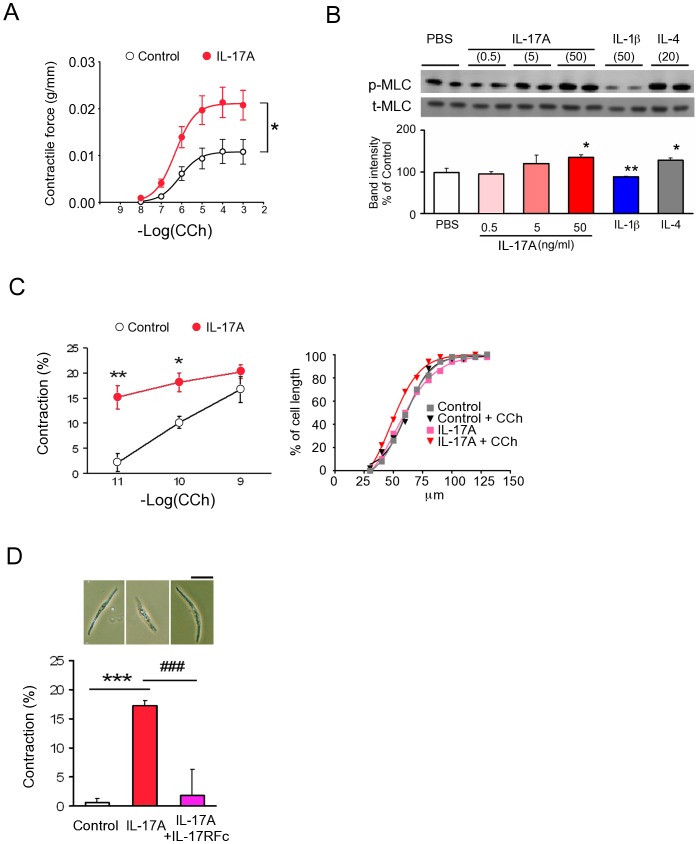
IL-17A induces hypercontractility of SI longitudinal MS and SMCs. (**A**) CCh-stimulated dose-response curves of MS contractility induced by IL-17A treatment for 3 days. CCh was added cumulatively (n = 9). (**B**) Immunoblot analysis of phosphorylated Ser19 of myosin light chain 2 (p-MLC) in samples of MS treated with or without IL-17A, IL-1β or IL-4 and quantitation of band intensity (n = 3∼4). The ratio of p-MLC to total MLC was calculated as described in Materials and Methods and normalized to untreated samples. N means the number of mice. Upper panels are representative images. (**C**) The Left panel shows CCh-stimulated dose-dependent changes of SMC contractility induced by IL-17A treatment for 24 hr. The respective concentration of CCh was added separately (n = 5). The right panel shows the distribution of cell length of 10^−11^ M CCh-stimulated SMCs with or without IL-17A treatment for 24 hr (n = 4). Y axis represents accumulating percentage of the number of the cells whose length are equal to, or smaller than, the length indicated in the x axis. The graph suggests that almost all of the analyzed SMCs responded to CCh stimulation. (**D**) Contractile response to CCh of SMCs treated with or without L-17A and/or IL-17R-Fc-Chimera (n = 6∼7). Upper panels are representative phase-contrast images. Scale bar, 50 µm. Numerical data represent means ± s.e.m. *P<0.05, **P<0.01 ***P<0.001 versus control/PBS-treated, ^###^P<0.001 versus IL-17A-treated. Repeated measure ANOVA (**A**), Student's t-test (**B-D**) with the closed testing procedure (**B**) and Bonferroni correction (**D**) for multiple comparisons.

### IL-17A induced hypercontractility in isolated LM strips and SMCs

Next we examined the effect of IL-17A on LM strip contractility *in vitro*. An LM strip in an organ bath that was repeatedly exposed to IL-17A for 3 days showed enhanced CCh-induced contraction (Figure 3A). Accordingly, immunoblot analysis of MS revealed that p-MLC, a surrogate marker as well as a key mechanistic factor of smooth muscle contraction [21], increased upon IL-17A treatment (Figure 3B). IL-1β (a relaxing agent) and IL-4 (a contracting agent) induced a decrease and increase in the level of p-MLC, respectively (Figure 3B). An enhancement of CCh-induced contraction was also found in the SMCs isolated from LM layers after incubation with IL-17A *in vitro* for 24 hr (Figures 3C). This effect was abrogated by co-treatment with IL-17R-Fc-Chimera (Figure 3D).

### IL-17A activated IκBζ signaling

**Figure 4:**
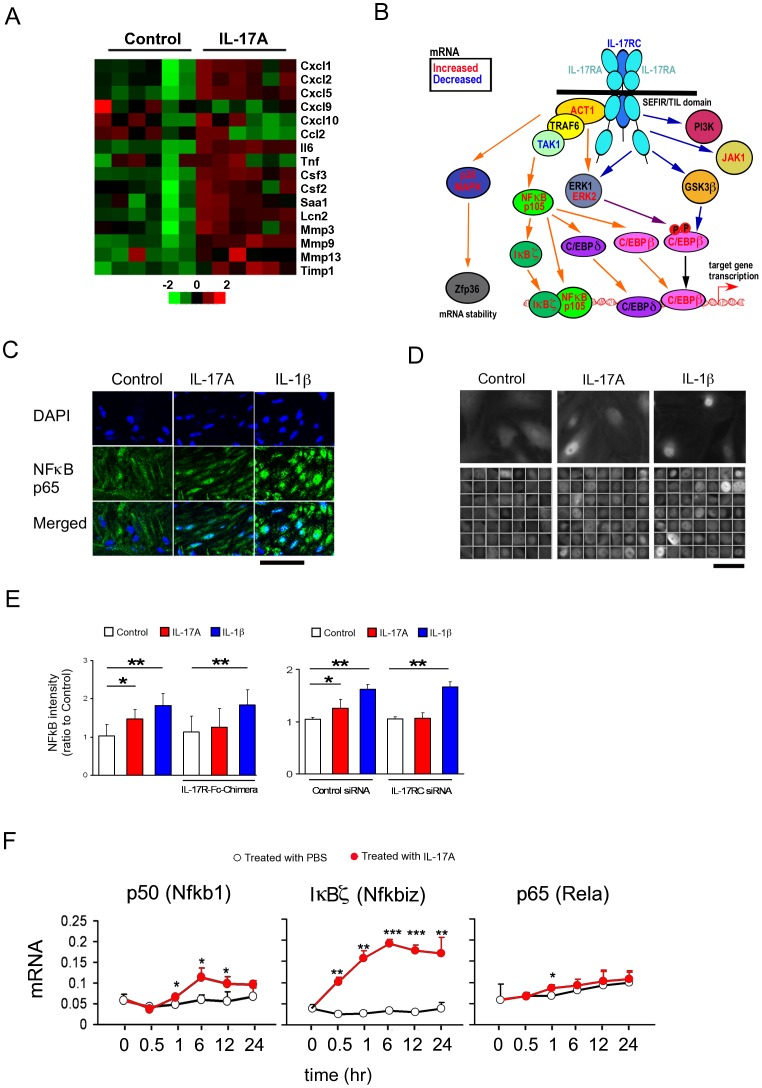
A possible involvement of the IκBζ pathway in IL-17A-induced hypercontractility. (**A**) Heatmap of genes expressed in MS after 24 h IL-17A treatment, assessed by microarray (n = 6). (**B**) Summary of changes of mRNA levels of the genes of IL-17A signalling cascade assessed by microarray (n = 6). Genes with significant changes in expression are coloured in red (increased) or blue (decreased). (**C**) Immunohistochemical staining for NFκB p65 protein in MS treated with or without IL-17A and IL-1β for 1 hr. Scale bars, 50 µm. (**D**) Immunofluorescence staining of NFκB p65 protein in primary cultured murine SMCs after 30 min treatment with IL-17A or IL-1β. Lower panels represent p65 immunosignal in the nuclei picked up automatically by the cell imaging system Celaview. Scale bar, 25 µm. (**E**) Relative intensity of NFκB p65 immunosignal accumulated in the nucleus treated with IL-17R-Fc-Chimera or IL17RC siRNA (n = 3∼6). (**F**) Time-dependent changes in mRNAs of NFκB proteins evaluated by real-time RT-PCR in mouse cultured SMCs (n = 6). Numerical data represent means ± s.e.m. *P<0.05, **P<0.01, ***p<0.001, Student's t-test (**E**, **F**) under the closed testing procedure for multiple comparison (**E**).

To investigate the molecular signalling, microarray analysis was performed on LM strip. The typical target genes of IL-17A were induced (Figure 4A), and IκBζ, a subset of the NFκB signalling pathway, was found to be upregulated after IL-17A treatment (Figure 4B). Accordingly, LM strips incubated with IL-17A for 1 hr showed nuclear translocation of NFκB p65 protein (Figure 4C). The effects of IL-17A on NFκB signalling were also observed in primary cultured murine SI SMCs (Figure S2A). Incubation with IL-17A rapidly (∼30 min) induced NFκB translocation in SMCs, similar to the effect elicited by IL-1β (Figure 4D). IL-17R-Fc-Chimera and IL-17RC siRNA abrogated the NFκB translocation induced by IL-17A but not by IL-1β (Figure 4E). Similar to the microarray data on LM strips, mRNAs of NFκB–related proteins, especially of IκBζ, was also potently induced in cultured SMCs (Figure 4F). These data suggest IL-17A-induced the activation of NFκB.

**Figure 5:**
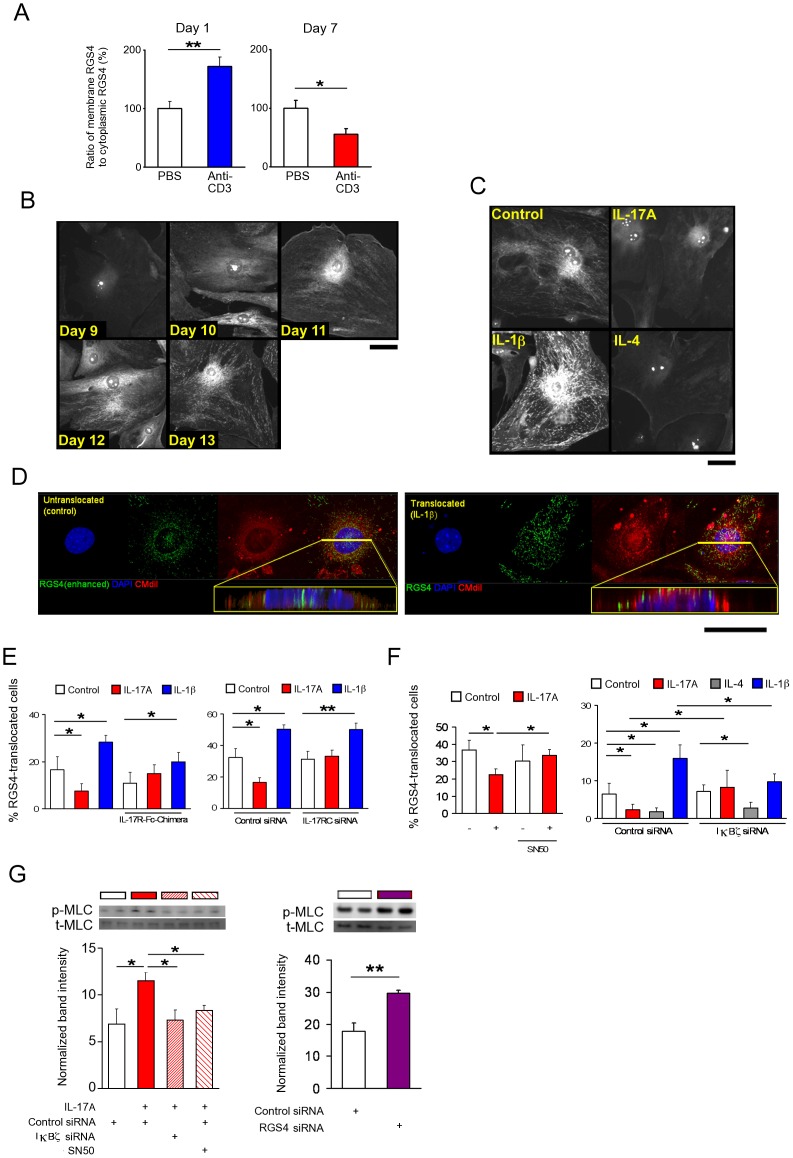
The effect of IL-17A on IκBζ - RGS4 signaling. (**A**) RGS4 activity in samples of MS isolated from the mice treated with PBS or αCD3. RGS4 activity was estimated as the ratio of membrane RGS4 to cytosolic RGS4 as described in Materials and Methods. In the inflammatory phase (Day 1) RGS4 activity was enhanced while in the recovery phase (Day 7) the activity was decreased (n = 8). (**B**) Immunofluorescence staining of RGS4 protein. The days from the beginning of the culture are indicated. Scale bar, 50 µm. (**C**) Immunofluorescence staining of RGS4 protein after treatment with IL-17A, IL-1β or IL-4 for 24 hrs. Scale bar, 50 µm. (**D**) Three dimensional reconstruction of laser confocal microscopic images of cultured SMCs treated with IL-1β (right panel) and control (left panel). Cross-sectional images are included. Scale bar, 50 µm. (**E**) RGS4 activity was assessed by calculating the ratio of the cells with punctate RGS4 immunosignals. The effect of IL-17A or IL-1β with or without L-17RFcChimera or IL-17RC siRNA was examined (n = 4∼5). (**F**) The effect of IL-17A, IL-1β, or IL-4 on RGS 4 activity was assessed with or without SN50, a p65 translocation inhibitor, or IκBζ siRNA (n = 4∼5). (**G**) Quantitation of p-MLC by immunoblot analysis. The ratio of p-MLC to total MLC was calculated as described in Materials and Methods. N means the number of wells prepared from a mixed single lot of SMCs derived from 24 mice. The experiment was repeated and essentially the same results were obtained. The left panel shows the effect of SN50 or IκBζ siRNAs on the IL-17A-induced increase in p-MLC (n = 3∼4). The right panel shows the effect of RGS4 siRNAs on the p-MLC (n = 3). Numerical data represent means ± s.e.m. *P<0.05, **P<0.01, Student's t-test under the closed testing procedure for multiple comparison (**A**, **E–F**).

### Analysis of RGS4 distribution in isolated LM strips from αCD3-treated mice

However, in GI SMCs, NFκB activation, which is typically triggered by IL-1β, results in the inhibition of ACh-stimulated initial contraction through upregulation in the expression of regulator of G protein signalling 4 (RGS4) [22]. RGS4 negatively regulates the strength and duration of signals mediated *via* Gα_q/11_ protein, which transmits the signals from ACh-stimulated muscarinic 3 receptor (M_3_R) to the downstream signalling cascade, e.g., activation of phospholipase C producing inositol 1,4,5–triphosphate and diacylglycerol [23]. These second messengers elicit the activation of protein kinase C, trigger an increase in [Ca^2+^]_i_ and drive the execution machinery of smooth muscle contraction consisting of myosin-actin interaction. Recruitment of RGS4 protein from the nucleus/cytosol to the M3R–Gα_q/11_ signalling complex residing in the plasma membrane is known to be critical for RGS4 functions. Therefore, quantification of the translocation of RGS4 from nucleus/cytoplasm to plasma membrane fraction has often been used to analyze the activity of this protein. In the present study, we firstly evaluated the distribution of RGS4 protein in isolated LM strips from αCD3-treated and PBS-treated mice. As shown in Figure 5A, in the inflammatory phase (Day 1), the ratio of RGS4 protein in the plasma membrane to that in the cytosol was larger in αCD3-treated mice. By contrast, this ratio was smaller in αCD3-treated mice in the recovery phase (Day 7). These data suggest that the suppressing effect of RGS4 on M3R–Gα_q/11_ signalling is increased in the inflammatory phase and decreased in the recovery phase.

### Analysis of RGS4 distribution in primary cultured SMCs

Next, we analyzed the intracellular distribution of RGS4 protein by immunofluorescent imaging in primary cultured SMCs (Figure 5B). SMCs began to express RGS4 protein from day 9, when the immunofluorescent signal was mainly confined to the nucleus and/or weakly diffused in the cytosol. However, over the following few days this signalling pattern gradually transformed to give bright punctate staining associated with the membrane. Indeed, the number of cells with punctate RGS4 staining increased spontaneously to 15–25% of SMCs by day 13. As shown in Figure 5C, IL-1β treatment on day 10 increased the number of cells with punctate RGS4 staining and augmented the signal intensity after 24 hr while IL-4 and IL-17 suppressed the occurrence of punctate RGS4 staining. In particular, IL-4 appeared to decrease the amount of RGS4 protein. Three dimensional reconstruction of RGS4 distribution by confocal laser microscopy revealed that the punctate RGS4 signals were located in and/or immediately beneath the plasma membrane, while the weak RGS4 signal was dispersed evenly throughout the nucleus and cytosol (Figure 5D). Thus the punctate distribution of RGS4 is a good indicator of RGS4 function [24]. Incubation with IL-17A reduced the number of SMCs with punctate RGS4 signals, and this was accompanied by a modest decrease in the level of RGS4. The IL-17A-induced downregulation of RGS4 function was inhibited by IL-17R-Fc-Chimera and IL-17RC siRNA, but had no effect on the IL-1β-induced increase in punctate RGS4 signals (Figure 5E). IL-1β-induced upregulation of RGS4 function is known to be mediated by NFκB signalling [25]. Therefore we examined whether NFκB signalling is involved in IL-17A-mediated downregulation of RGS4 function. The effect of IL-17A was inhibited by pre-treatment with SN50, a competitive inhibitor of nuclear NFκB p65 (RelA) translocation as well as siRNA to IκBζ (Figure 5F). Surprisingly, the upregulation of RGS4 by IL-1β was also inhibited by IκBζ siRNA. Although the IL-4 was found to downregulate RGS4 function akin to IL-17, the effect of IL-4 on RGS4 was unaffected by IκBζ siRNA treatment (Figure 5F). IL-17A treatment increased p-MLC, which was abrogated by IκBζ siRNA and SN50, while RGS4 siRNA treatment increased the amount of p-MLC (Figure 5G).

### Involvement of MAPKs for determination of the direction of contractile response

**Figure 6:**
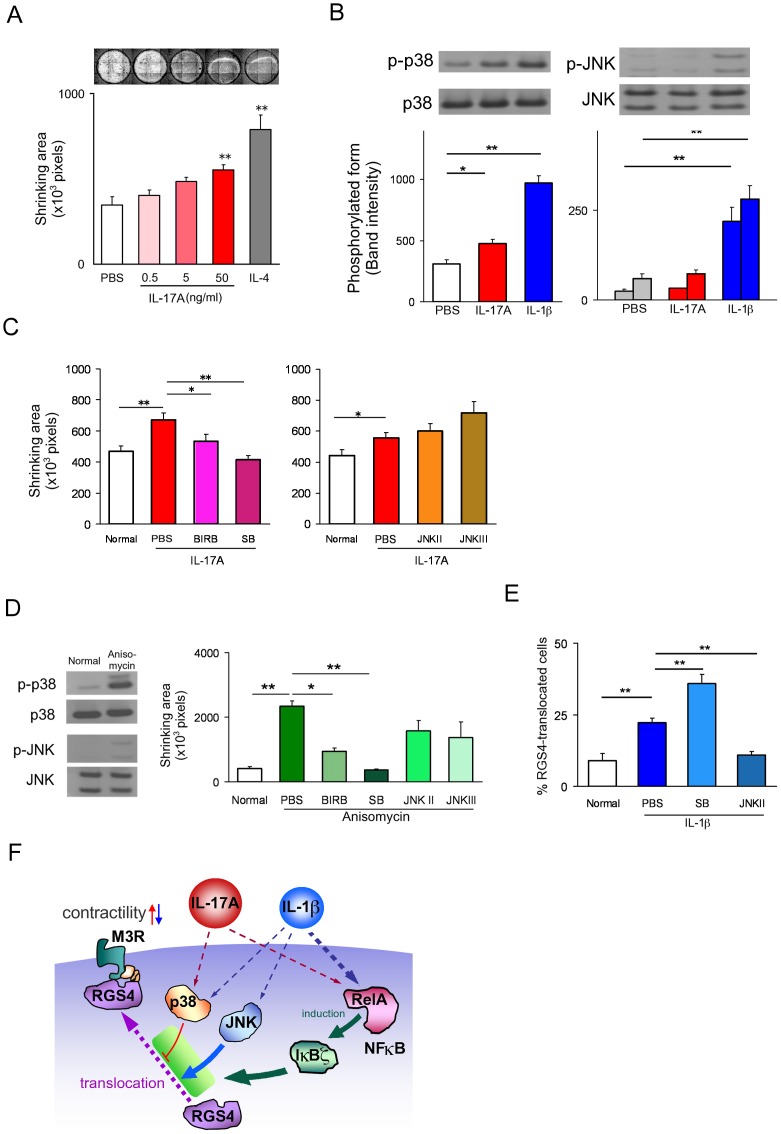
Involvement of p38MAPK activation in IL-17A-mediated hypercontractility in primary cultured murine SI SMCs. (**A**) Contractility assay of IL-17A-treated SMCs. SMCs were cultured with IL-17A or IL-4 for 4 days using the temperature-responsive cell culture surface, UpCell plates. After addition of 10^−11^ M CCh, the cell layers became detached from the well during incubation at room temperature. Cell shrinkage was calculated by measuring the area of cell layers (n = 3∼6). Upper panels are representative images. (**B**) Quantitation of activation of p38MAPK (left panel) and JNK (right panel) by immunoblot analysis. The amount of phosphorylated form of p38MAPK (p-p38) and JNK (p-JNK) was measured (n = 4). (**C**) Contractility assay of IL-17A-treated SMCs. The effects of p38MAPK inhibitors (left panel) and JNK inhibitors (right panel) are shown. SB203580 (SB, 1 µM), BIRB796 (BIRB, 10 nM), SP600125 (JNK II, 100 nM) and JNK inhibitor III (JNK III, 100 nM) were added 15 min before addition of IL-17A (50 ng/ml). Contractility was measured as described in Fig. 6A (n = 8). (**D**) Effect of MAPK inhibitors on on anisomycin-induced contractility. The activation of p38MAPK by anisomycin was demonstrated by the representative images of the immunoblot in the left panel. The right panel shows the effects of p38MAPK and JNK inhibitors on anisomycin-induced contractility. Contractility was measured the day after addition of anisomycin (n = 4). (**E**) The effects of p38MAPK and JNK inhibitors on IL-1β-induced RGS4 translocation. RGS4 activity was evaluated by Celaview analysis as described in Fig. 5D (n = 4). Numerical data represent means ± s.e.m. *P<0.05, **P<0.01, Student's t-test under the closed testing procedure for multiple comparison (**A–E**). (**F**) Scheme of IL-17A and IL-1β signalling leading to hyper- and hypo-contractility induced by IL-17A and IL-1β, respectively. IL-17A induces p38 MAPK phosphorylation and NFκB activation (i.e., translocation of p65 RelA protein into the nucleus) independently within 15 min. Inhibition of p38 MAPK activation had no effect on RelA translocation and conversely, inhibition of NFκB activation had no effect on MAPK phosphorylation (data not shown). The expression of IκBζ, which is virtually absent without NFκB activation was massively induced after 30 min. The decrease in translocation of RGS4 protein to the cell membrane, where RGS4 suppresses muscarinic receptor-mediated signalling via interaction with G proteins coupled to muscarinic receptor, was observed between day 1 and day 4. A significant hypercontractility of SMCs began to be detected after day 2 and continued through to day 4. Inhibition of p38MAPK and NFκB/IκBζ activation both suppressed IL-17-induced hypercontractility and the changes of RGS4 translocation. By contrast, IL-1β activates JNK as well as NFκB/IκBζ and p38MAPK. Inhibition of NFκB/IκBζ and JNK abrogated the effect of IL-1β while p38MAPK inhibition enhanced it. Thus the balance in the relative activity levels of JNK and p38MAPK is critical for determining the direction of contractility. NFκB-IκBζ signalling regulates the movement of MAPK-triggered molecular events toward/against hypercontractility of SI SMC.

**Figure 7:**
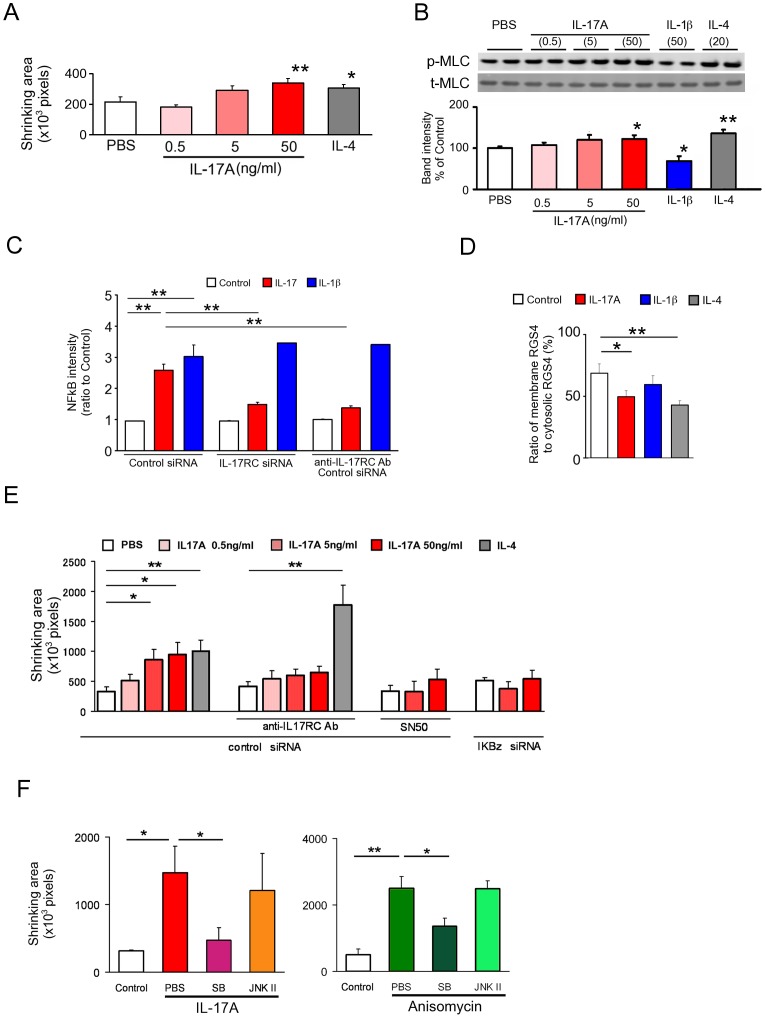
IL-17A-induced hypercontractility and its signal transduction in cultured human colonic SMCs. (**A**) Contractility assay of IL-17A-treated human SMCs. SMCs were cultured with IL-17A and IL-4 for 5 days and contractility was evaluated as described in Figure? 6A. (**B**) Immunoblot analysis of p-MLC and quantitation of band intensity in samples of colonic SMCs treated with or without IL-17A, IL-1β or IL-4 (n = 6). The cells were treated with 10^−11^ M CCh for 3 min. The ratio of p-MLC to total MLC was calculated as described in Materials and Methods and normalized to untreated samples. N refers to the number of experiments. Upper panels are representative images. (**C**) Relative intensity of NFκB p65 immunosignal accumulated in the nucleus was measured in cultured colonic SMCs after 30 min treatment with PBS, IL-17A or IL-1β in the absence or presence of anti-IL-17RC antibody or siRNAs to control and IL-17RC. (**D**) The effect of IL-17A and IL-1β on RGS 4 activity in human SMCs on day 4 was assessed (n = 4). RGS4 activity was evaluated as described in Materials and Methods. (**E**) Effect of anti-IL-17RC antibody, SN50 and IκBζ siRNA on IL-17A-induced contractility in human SMCs. Contractility was measured on day 4 (n = 3–6) as described in Fig.6A. (**F**) The effects of MAPK inhibitors on IL-17A- and anisomycin-induced hypercontractility on day 4 (n = 3–6). The left and right panels show the effects MAPK inhibitors on IL-17A-, and anisomycin-induced contractility, respectively. Contractility was measured as described in Fig. 6A (n = 3). Numerical data represent means ± s.e.m. *P<0.05, **P<0.01, Student's t-test under the closed testing procedure for multiple comparison (**A–F**).

In order to obtain more direct evidence, we have developed an *in vitro* contractility assay of primary cultured SMCs using temperature-responsive plates. IL-17A-induced hypercontractility was demonstrated during day 2 to 4 (Figure S2B, Figure 6A), which was abrogated by NFκB inhibition (Figure S2C). The decrease in RGS4 induced by IL-17A was also maintained on day 4 (Figure S2D). Next, activation of various MAPKs was screened to investigate the possible mechanisms producing the opposing biochemical activities of IL-17A and IL-1β. We have found that IL-17A activates p38MAPK only while IL-1β activates p38MAPK and JNK1/JNK2 (Figure S2E, Figure 6B). The inhibition of p38MAPK abrogated IL-17A induced hypercontractility, while inhibition of JNK did not (Figure 6C). Anisomycin, a p38MAPK activator, also induced hypercontractility which was p38MAPK-mediated but JNK-unmediated (Figure 6D). IL-1β-induced RGS4 translocation was abrogated by JNK inhibitors and enhanced by p38MAPK inhibitors (Figure 6E). These data suggest that a balance between the relative activities of p38MAPK and JNK may determine the direction of the biological effect i.e., excess p38MAPK activity leads to hypercontractility whereas excess JNK activity leads to hypocontractility. Taken together, these findings imply that IL-17A enhances muscle contractility *via* downregulation of RGS4 translocation, which is modulated by p38MAPK and fuelled by NFκB- IκBζ (Figure 6F). Finally, we examined the effect of IL-17A on cultured human colonic SMCs (Figure S3A) and essentially the same results as those for murine SMCs were obtained, such as dose-dependent cell shrinking (Figures 7A), MLC phosphorylation (Figure 7B), NFκB activation (Figure 7C, Figure S3B), RGS4 suppression (Figure 7D), and involvement of IκBζ (Figure 7D, Figure S3C) and p38 MAPK (Figure 7E, Figure S3D).

## Discussion

The present study indicates that IL-17A can induce hypercontractility during the healing stage of T cell-mediated intestinal inflammation through RGS4 signalling in SMCs. Although the present enteritis model does not simulate the pathophysiology of clinical GI disorders such as celiac disease, IBD and IBS, our findings may provide a novel point of view for evaluating the role of T cells and IL-17A in GI disorders.

T cell activation has been observed in a variety of GI disorders. Celiac disease is a T-cell-mediated autoimmune enteropathy due to ingested wheat gluten and related proteins [26]. Altered motility in various GI regions has been reported, not only in untreated celiac disease patients but also in patients on a gluten-free diet who showed unequivocal clinical improvement due to changes in their diet. These reports indicate that T cell activation may induce GI motor disorders in human. However, the reported abnormalities in the small intestines were predominantly the hypomotility concomitant with intestinal inflammation [27]–[29] while accelerated transit was observed only in the colon. Similarly, it has been reported that IBD patients display hypomotility or reduced contractility in the small intestines [30]–[32]. A considerable percentage of IBD patients develop IBS symptoms including diarrhoea especially during remission [33]. However, whether a real hypermotility or power propulsion response could account for diarrhoea in such patients is still uncertain. For IBS patients, although they do not manifest histological aberration, there is now good evidence for low grade inflammation and immune activation [34], [35] including the increase in the intestinal level of CD3, CD4 and CD8 cells [36]–[38]. Accelerated transit in the colon and small intestines has been frequently observed in IBS patients [39]–[42]. Furthermore, a recent study addresses the possibility that enhanced SI transit could be clinically translated into the generation of symptoms in a subset of IBS patients [29]. Thus a relationship between T cell activation and intestinal hypermotility has been suggested in a wide variety of GI diseases.

The importance of Th17 cells in the pathogenetic mechanisms of IBD (and possibly of celiac disease) has been suggested by numerous studies especially of population genetics and animal disease models [43]–[49]. However, the failure of anti-IL17A antibody therapy for Crohn's disease in phase II clinical trials has questioned the postulated role of IL-17A in a pathogenetic mechanism of inflammatory GI diseases [50], [51]. Furthermore, evidence suggesting the involvement of IL-17/23 axis in the pathophysiology of IBS is still lacking. Thus the pathophysiological significance of IL-17A in GI diseases has not been established.

In addition to the ambiguous role of IL-17A in GI diseases, coexistence of various cytokines makes it difficult to delineate the effect of IL-17A on the motility in GI diseases. Th2 cytokines such as IL-4 and IL-13, are known to induce hypercontractility [12]–[14] while Th1 and proinflammatory cytokines (e.g., IL-1β, TNF-α, and IFN-γ) induce hypocontractility [8]–[10]. Therefore, the final outcome during the cytokine surge induced by active inflammation is a composite of the multiple effects elicited by numerous cytokines resulting in the masking of the effect brought on by IL-17A. In the present study, it appeared that IL-17A began to exert its effect on contractility after the termination of the cytokine surge when the significant increase in IL-17A protein still persisted. It should be noted that SI is an extraordinarily specialized locus for control of Th17 cell pathogenicity for systemic and peripheral chronic inflammatory disorders of various organs [52]. In the report, T cell receptor activation resulted in the prominent proliferation and/or influx of IL-17A-expressing T cells in the SI (in essentially the same experimental system using intraperitoneal injection of αCD3, 50–80% of CD4^+^TCRαβ^+^ T cells in the duodenum express IL-17A). Thus a moderate level of IL-17A in the absence of apparent intestinal inflammation might induce hypermotility in PI-IBS and IBD-IBS. Investigation of cytokine profiles after the resolution of, or during the absence of, evident inflammation may help to clarify this point.

The direct induction of SMC hypercontractility by IL-17A has also been reported in allergen-induced airway hyper-responsiveness without any obvious effects on airway inflammation [53]. The authors showed that the enhancement of hypercontractility by IL-17A in airway-hyper-responsiveness involves NFκB signalling whose downstream cascade consists of a ras homolog gene family, member A (RhoA) and Rho-associated, coiled-coil–containing protein kinase 2 (ROCK2). This addresses the assumption that the IL-17A-induced hypercontractility might be involved in the disorders of various organs. Investigations into the possible role of IL-17A in the contractility of SMCs of other organs, such as the uterus, blood vessels, urinary bladder and so forth, may open a new approach to treatment of diseases related to abnormal contractility of SMCs e.g., hypertension, asthma, pollakiuria, subarachnoid hemorrhage and threatened abortion.

The present study investigated the mechanism by which IL-1β and IL-17A exert the opposite effect on SMC contractility. While IL-1β appears to reduce contractility in intestinal tissues and SMCs [54], [55], many reports have shown that this cytokine enhances contractility in lung tissues and airway SMCs [56], [57]. IL-1β has been reported to activate NFκB signaling and inhibition of NFκB signaling, which abrogates the IL-1β-induced alteration of contractility in both types of SMCs [25], [58]. Furthermore, in the present study, IL-1β and IL-17A exert the opposite effect on SMC contractility in the same intestinal SMC preparation both in an NFκB-dependent manner. These data suggest additional signaling(s) is/are involved in the determination of direction of SMC contractility in addition to NFκB signaling. Moreover, the present results indicate a possible involvement of MAPKs; the NFκB-triggered effect on contraction was correlated with an increase in p38MAPK activity but to a decrease in JNK activity. This is intriguing because it has been reported that the pro-asthamtic contraction of airway SMCs by lipopolysaccharide is induced by activation of ERK1/2 and counteracted by p38MAPK [59]. Along these lines, Murthy and colleagues have been investigating the possible involvement of NFκB, MAPKs and RGS4 using rabbit colonic smooth muscle cells [25], [60], [61]. They reported the activation of ERK1/2 and p38 MAPK enhances, and PI3K/Akt/GSK3β signaling reduces, the IL-1β-induced, NFκB-mediated RGS4 upregulation in rabbit colonic SMCs [60]. Furthermore, they also recently reported that JNK activation decreases RGS4 expression in the presence or absence of IL-1β stimulation [61]. Their results are opposite to those of the present study in terms of the direction of action of P38 MAPK and JNK, and differ in the presence or absence of ERK1/2 activation (we have not observed evident ERK1/2 activation by IL-1β). The apparent discrepancy may be due to differences in the sources of intestinal preparation (rabbit colon vs mouse small intestine/human colon) or experimental conditions (methods of preparation and culture, etc.). Regardless, these findings suggest the balance of MAPK activities may be critically involved in the determination of the direction of contractile response in lung and intestinal SMCs. Further investigations into the possible role of MAPKs in the contractility of SMCs from other organs will greatly assist in our understanding of SMC biology.

In summary, the present study indicates that IL-17A induces hypermotility by enhancing contractility of SMC and clarified its signalling cascade, which consists of IκBζ-RGS4 in crosstalk with p38MAPK. Although the relevance of these findings to human GI diseases is unclear, our results suggest it may be important to investigate the possible role of IL-17A in GI pathophysiology from a novel, previously unpostulated context.

## Supporting Information

Figure S1 **Profiles of αCD3-induced enteropathy of wild-type and IL-17A KO mice.** (**A**) body weight, (**B**) histology, (**C**) tissue cytokine mRNAs measured by real-time RT-PCR (top) and tissue cytokine proteins measured by Bio-plex (bottom). Wild-type mice treated with 12.5 µg/body of αCD3 resulted in massive apoptosis in the small intestine with the least influence on the proinflammatory cytokine levels in the blood as previously reported by ourselves [19]. Mice developed diarrhoea within 4 hours. Body weight loss was observed from day 1 to day 3, although the weight started to recover from day 3 onwards as shown in Figure S1A (n = 6–20). Macroscopically, there was fluid accumulation in the small intestine of αCD3-treated mice. The small-intestinal mucosa of αCD3-treated mice was characterised by reduced villous height, increased thickness of the crypt region and infiltration of inflammatory cells. The histological features returned to normal by day 7. No gross histological damage to the circular or longitudinal muscle layers was observed (Figure S1B; Scale bar, 200 µm). With the exception of IL-23, all mRNA of tested cytokines were rapidly induced and significant elevation for several cytokines above their normal level was observed e.g., IL-1β, TNF-α, IFN-γ and IL-17A (Figure S1C; n = 6). The elevation in the level of cytokine proteins was transient, although significantly higher levels of IL-17A persisted until day 7. The changes in body weight and cytokine profiles are essentially the same between wild-type mice and IL-17A KO mice. Examination by a professional histologist in a blind manner found no evident difference between wild type and IL-17A KO in the degree and profile of αCD3-induced inflammation (data not shown; n = 5). These results strongly suggest no significant variation between the enteropathy in wild-type mice and that of IL-17A KO mice. Data represent means ± s.e.m. *P<0.05, **P<0.01 versus (Control/PBS-treated), Student's t-test (**C**).
(TIF)

Figure S2 **Murine LM was digested in the buffer containing 0.1% type II collagenase and 0.1% soy bean trypsin inhibitor (Sigma-Aldrich) and the dispersed cells were plated in type IV-collagen coated plates in HuMedia-SG2 (Kurabo, Osaka, Japan).** After 9 days of culture, medium was replaced to non-serum medium M199 containing antibiotics-antimycotics (Sigma-Aldrich). Immunocytochemistry using antibodies to PGP9.5, GFAP, p75-NGF-receptor, F4/80, CD117, Pan-Neuro and α-smooth muscle actin revealed that the purity of cultured SMCs were >95%. (**A**) Immunostaining by αα-smooth muscle actin (αSMA) of murine SI SMCs is shown. The figure shows a well differentiated SMC (with large nucleus and potent immunostaining by αSMA) and differentiating SMCs (with small nucleus and weak immunostaining by αSMA). Scale bar, 50 µm. (**B**) Contractility assay of IL-17A-treated murine SMCs on day 2. SMCs were cultured with IL-17A, IL-4 or anisomycin for 2 days and contractility was evaluated as described in Figure 6A. (**C**) Effect of NFκB inhibitor on IL-17A-induced contractility in murine SMCs. An NFκB inhibitor type IV (1 µM, Calbiochem) was added 15 min before IL-17A addition. Contractility was measured on day 4 (n = 3–6) as described in Fig. 6A. (**D**) The effect of IL-17A and IL-1β on RGS 4 activity in murine SMCs on day 4 was assessed (n = 4). RGS4 activity was evaluated as described in Figure 7D. (**E**) Screening of MAPK activities induced by IL-17A or IL-1β. Murine SMCs cultured with cytokines for 4 days were lysed and activities of 24 MAPKs were measured using a ProteomeProfiler kit (R&D Systems). Numerical data represent means ± s.e.m. *P<0.05, **P<0.01, Student's t-test under the closed testing procedure for multiple comparison (**B–D**).
(TIF)

Figure S3 **Human colonic SMCs were obtained from ScienCell Research Laboratories and cultured according to the supplier's protocol.** (**A**) Immunostaining by α-smooth muscle actin (αSMA) of human SI SMCs is shown. (**B**) Immunofluorescence staining of NFκB p65 protein in primary cultured human SMCs after 30 min treatment with IL-17A, IL-1β or IL-4. Scale bar, 50 µm (**C**) The effect of IL-17RC and IκBζ siRNAs on p-MLC in human colonic SMCs treated with IL-17A, IL-1β and IL-4 (n = 4). p-MLC was evaluated as described in Figure 5F. (**D**) Screening of MAPK activities induced by IL-17A or IL-1β. Human SMCs cultured with cytokines for 4 days were lysed and activities of 24 MAPKs were measured using a ProteomeProfiler kit. Numerical data represent means ± s.e.m. *P<0.05, **P<0.01, Student's t-test under the closed testing procedure for multiple comparison (**C**).
(TIF)
