# Supplementary figures and images for: Involvement of Interleukin-17A-Induced Hypercontractility of Intestinal Smooth Muscle Cells in Persistent Gut Motor Dysfunction
Source: PLoS One. 2014 May 5;9(5):e92960. doi: 10.1371/journal.pone.0092960 (PMC4010403; doi:10.1371/journal.pone.0092960)

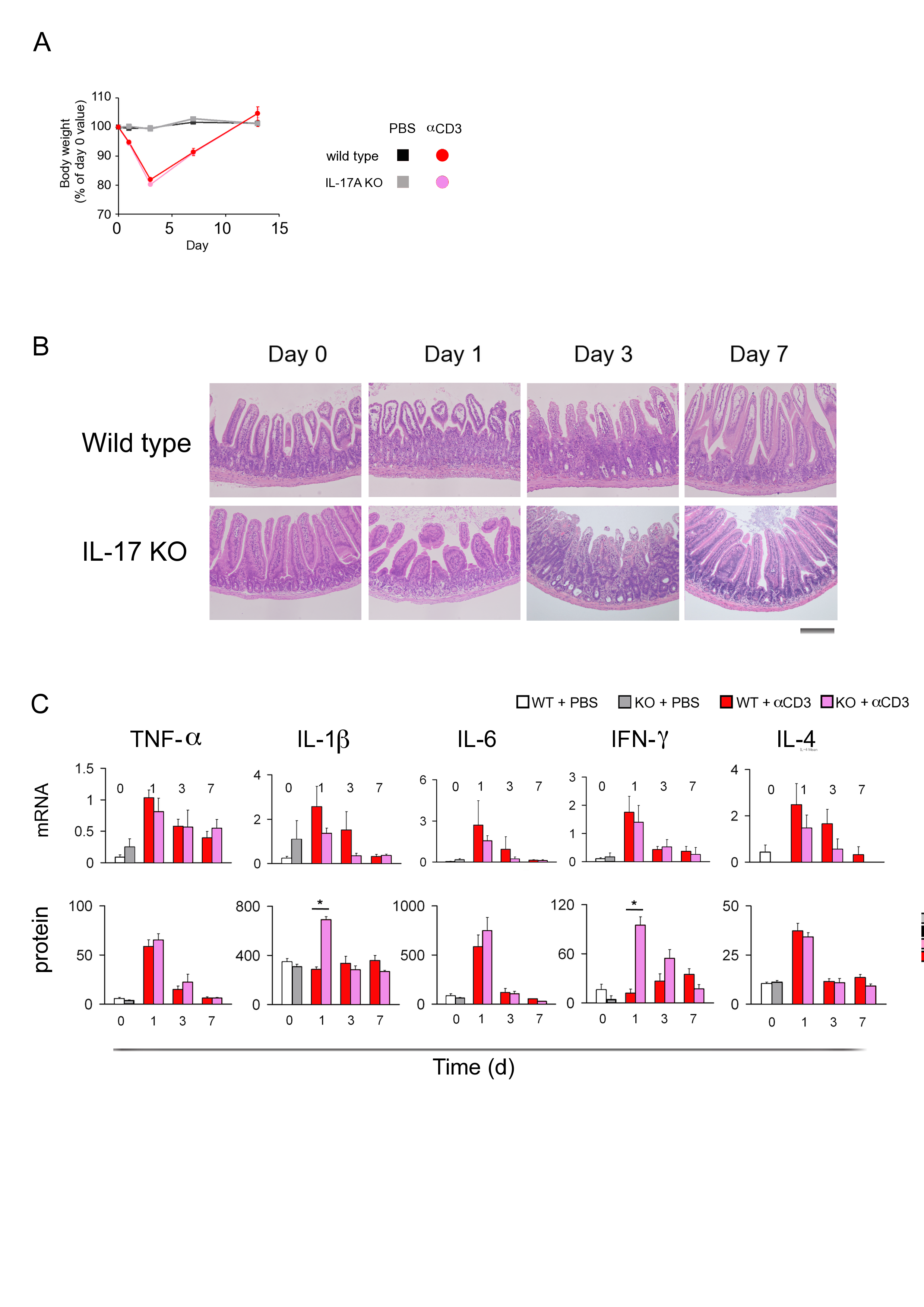

Supplement: Figure S1 — Profiles of αCD3-induced enteropathy of wild-type and IL-17A KO mice. (A) body weight, (B) histology, (C) tissue cytokine mRNAs measured by real-time RT-PCR (top) and tissue cytokine proteins measured by Bio-plex (bottom). Wild-type mice treated with 12.5 µg/body of αCD3 resulted in massive apoptosis in the small intestine with the least influence on the proinflammatory cytokine levels in the blood as previously reported by ourselves [19]. Mice developed diarrhoea within 4 hours. Body weight loss was observed from day 1 to day 3, although the weight started to recover from day 3 onwards as shown in Figure S1A (n = 6–20). Macroscopically, there was fluid accumulation in the small intestine of αCD3-treated mice. The small-intestinal mucosa of αCD3-treated mice was characterised by reduced villous height, increased thickness of the crypt region and infiltration of inflammatory cells. The histological features returned to normal by day 7. No gross histological damage to the circular or longitudinal muscle layers was observed (Figure S1B; Scale bar, 200 µm). With the exception of IL-23, all mRNA of tested cytokines were rapidly induced and significant elevation for several cytokines above their normal level was observed e.g., IL-1β, TNF-α, IFN-γ and IL-17A (Figure S1C; n = 6). The elevation in the level of cytokine proteins was transient, although significantly higher levels of IL-17A persisted until day 7. The changes in body weight and cytokine profiles are essentially the same between wild-type mice and IL-17A KO mice. Examination by a professional histologist in a blind manner found no evident difference between wild type and IL-17A KO in the degree and profile of αCD3-induced inflammation (data not shown; n = 5). These results strongly suggest no significant variation between the enteropathy in wild-type mice and that of IL-17A KO mice. Data represent means ± s.e.m. *P<0.05, **P<0.01 versus (Control/PBS-treated), Student's t-test (C). (TIF) [file pone.0092960.s001.tif]

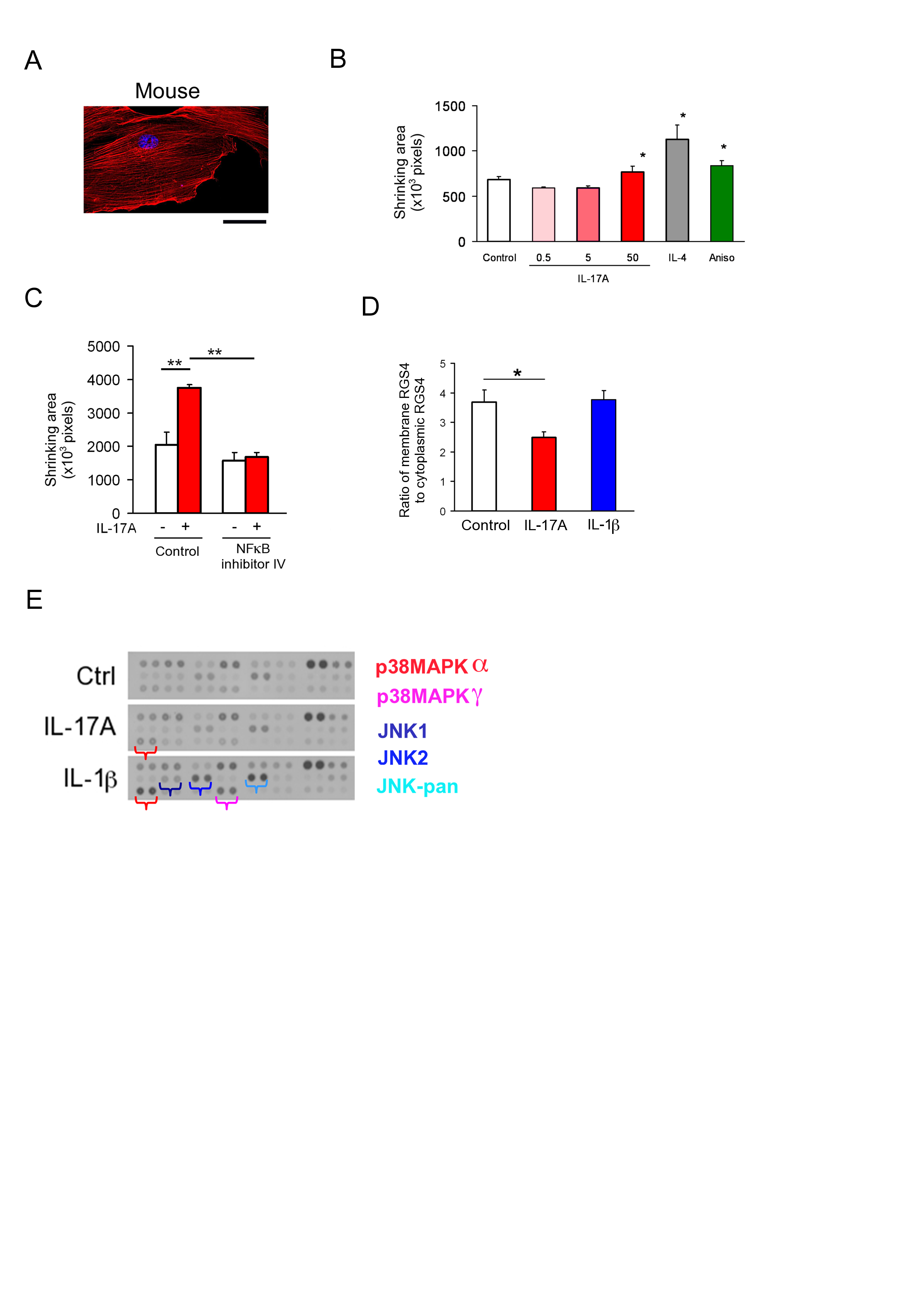

Supplement: Figure S2 — Murine LM was digested in the buffer containing 0.1% type II collagenase and 0.1% soy bean trypsin inhibitor (Sigma-Aldrich) and the dispersed cells were plated in type IV-collagen coated plates in HuMedia-SG2 (Kurabo, Osaka, Japan). After 9 days of culture, medium was replaced to non-serum medium M199 containing antibiotics-antimycotics (Sigma-Aldrich). Immunocytochemistry using antibodies to PGP9.5, GFAP, p75-NGF-receptor, F4/80, CD117, Pan-Neuro and α-smooth muscle actin revealed that the purity of cultured SMCs were >95%. (A) Immunostaining by αα-smooth muscle actin (αSMA) of murine SI SMCs is shown. The figure shows a well differentiated SMC (with large nucleus and potent immunostaining by αSMA) and differentiating SMCs (with small nucleus and weak immunostaining by αSMA). Scale bar, 50 µm. (B) Contractility assay of IL-17A-treated murine SMCs on day 2. SMCs were cultured with IL-17A, IL-4 or anisomycin for 2 days and contractility was evaluated as described in Figure 6A. (C) Effect of NFκB inhibitor on IL-17A-induced contractility in murine SMCs. An NFκB inhibitor type IV (1 µM, Calbiochem) was added 15 min before IL-17A addition. Contractility was measured on day 4 (n = 3–6) as described in Fig. 6A. (D) The effect of IL-17A and IL-1β on RGS 4 activity in murine SMCs on day 4 was assessed (n = 4). RGS4 activity was evaluated as described in Figure 7D. (E) Screening of MAPK activities induced by IL-17A or IL-1β. Murine SMCs cultured with cytokines for 4 days were lysed and activities of 24 MAPKs were measured using a ProteomeProfiler kit (R&D Systems). Numerical data represent means ± s.e.m. *P<0.05, **P<0.01, Student's t-test under the closed testing procedure for multiple comparison (B–D). (TIF) [file pone.0092960.s002.tif]

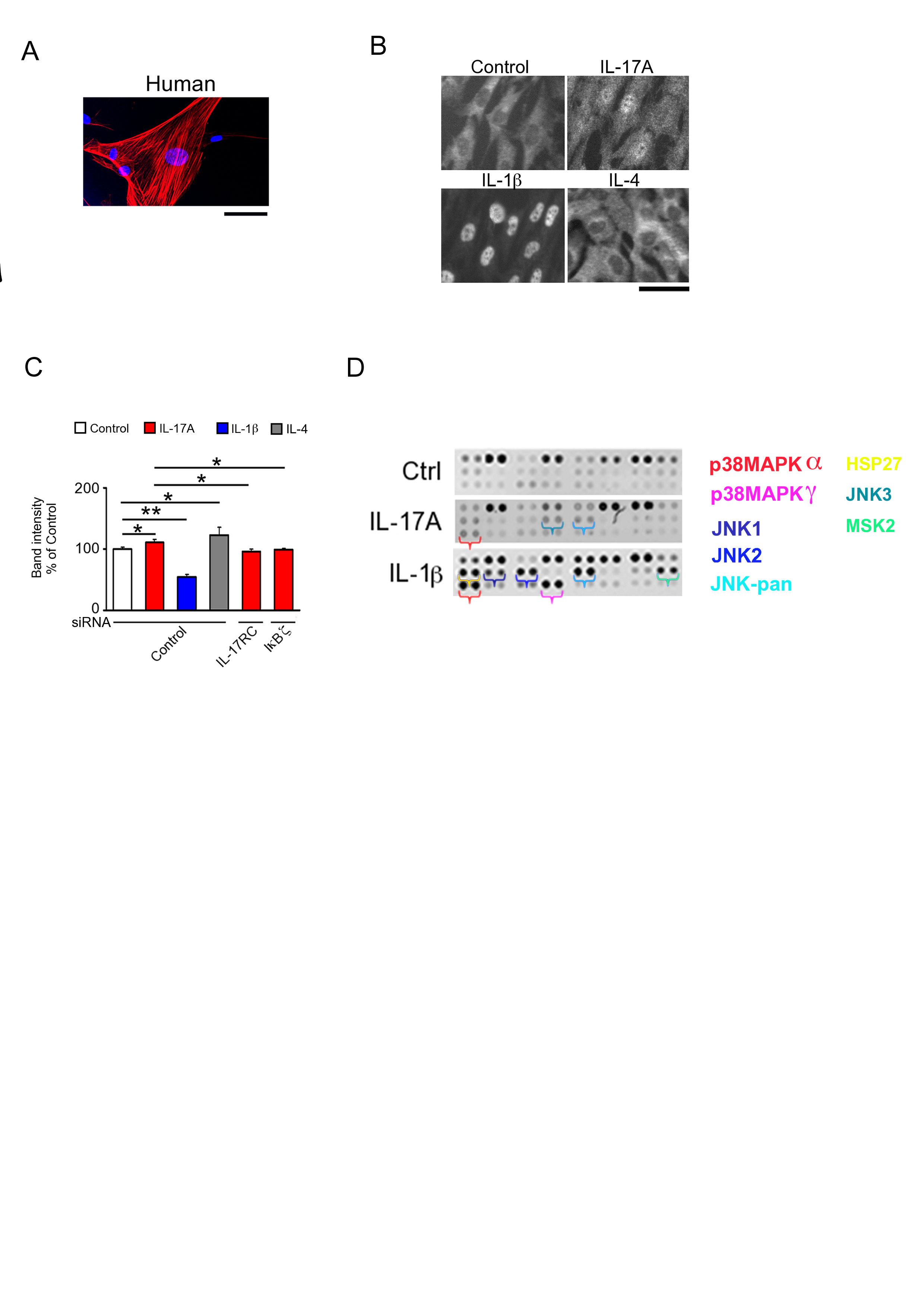

Supplement: Figure S3 — Human colonic SMCs were obtained from ScienCell Research Laboratories and cultured according to the supplier's protocol. (A) Immunostaining by α-smooth muscle actin (αSMA) of human SI SMCs is shown. (B) Immunofluorescence staining of NFκB p65 protein in primary cultured human SMCs after 30 min treatment with IL-17A, IL-1β or IL-4. Scale bar, 50 µm (C) The effect of IL-17RC and IκBζ siRNAs on p-MLC in human colonic SMCs treated with IL-17A, IL-1β and IL-4 (n = 4). p-MLC was evaluated as described in Figure 5F. (D) Screening of MAPK activities induced by IL-17A or IL-1β. Human SMCs cultured with cytokines for 4 days were lysed and activities of 24 MAPKs were measured using a ProteomeProfiler kit. Numerical data represent means ± s.e.m. *P<0.05, **P<0.01, Student's t-test under the closed testing procedure for multiple comparison (C). (TIF) [file pone.0092960.s003.tif]
